# Effects of administering the maternal bovine appeasing substance on growth performance, cortisol level and carcass characteristics of finishing feedlot cattle

**DOI:** 10.1016/j.vas.2025.100457

**Published:** 2025-04-26

**Authors:** Shea J. Mackey, Reinaldo F. Cooke, Izadora S. de Souza, Autumn T. Pickett

**Affiliations:** Department of Animal Science - Texas A&M University, College Station, TX 77845, USA

**Keywords:** Bovine appeasing substance, Feedlot, Finishing cattle, Productivity, Stress

## Abstract

This experiment evaluated physiological and performance responses of feedlot steers administered the maternal bovine appeasing substance (**mBAS**) during the finishing period (day 0 to 178). Steers used in this experiment were the same used to evaluate mBAS during the receiving period whose results have been published (Pickett et al., 2024). After the receiving period, steers were fed a growing diet for 65 days before the finishing period (without mBAS administration). On day 0, steers were assigned to topical administration (10 mL) of: 1) mineral oil on day 0 and 88 (**CON**), or 2) mBAS on day 0 and 88. Steers that were administered mBAS during the receiving period were also administered mBAS during the finishing period, and the same process applied to CON steers. Cortisol concentrations were measured in tail-switch hair on days 0 and 15. Steers received a growth-promoting reimplant on day 88. Total cattle used since the receiving period was 120 Angus steers, which were assigned in 10 drylot pens (12 steers/pen, 5 pens/treatment). Steers were slaughtered on day 179, and carcass variables were measured. Feed intake did not differ (*P* = 0.57) between treatments, but average daily gain was increased by 5.6 % (*P* < 0.04) in mBAS steers, improving their gain:feed by 7.6 % (*P* < 0.01). No treatment effects were noted (*P* ≥ 0.24) for carcass weight and variables. Collectively, mBAS administration to feedlot steers at the beginning of the finishing period and at reimplant improved growth rates without altering feed intake, suggesting the use of mBAS to promote productive efficiency of feedlot systems.

## Highlights

• Feedlot cattle are exposed to several stressors during the finishing period.
• These stressors can impact productivity of finishing cattle.
• The maternal bovine appeasing substance has the potential to alleviate stress.
• Applying this substance improved performance and efficiency of finishing cattle.

## 1 Introduction

Feedlot cattle are exposed to stressors during the finishing period that affect their productivity and carcass quality (Cooke et al., 2025; Kumar et al., 2023; Mackey, 2024a). Examples include road-transport, handling for initial processing, and exposure to novel environments and diets (Duff & Galyean, 2007). Processing finishing cattle to administer a terminal growth-promoting implant also yields stress consequences that reduce feed intake (Cooke et al., 2025; Smith et al., 2020; Sperber et al., 2024). Despite recent advances to mitigate management-induced stress during feedlot receiving (Galyean et al., 2022), research-based strategies with the same purposes are warranted for finishing cattle.

The maternal bovine appeasing substance (**mBAS**) alleviates the physiological consequences of stress (Colombo et al., 2020; Fonseca et al., 2021), most likely by modulating neuroendocrine responses via the vomeronasal organ (Cappellozza & Cooke, 2022). The mBAS includes a mixture of fatty acids that replicate the composition of the original bovine appeasing pheromone, which promotes cow-calf bonding via recognition of maternal odors by the offspring (Cappellozza & Cooke, 2022). In a companion manuscript (Pickett et al., 2024), mBAS administration to high-risk steers improved their immunocompetence, reduced mortality, and enhanced pen-based productivity during a 60-day feedlot receiving period. Perhaps additional applications of mBAS during the finishing period, including when cattle receive a growth-promoting reimplant, yield further benefits to feedlot cattle welfare and productivity. Cooke et al. (2025) reported that mBAS administration to finishing Angus steers at feedlot arrival and reimplant (75 days after arrival) improved growth rate, feed intake, and carcass quality. Therefore, we hypothesized that applying mBAS during stressful events within the finishing period (upon arrival and reimplanting; Cooke et al., 2025) will further improve growth rates and production efficiency in cattle administered mBAS during the receiving period.

To test this hypothesis, a growth performance trial was carried out with steers from Pickett et al. (2024) that were fed finishing diets for 178 days. Steers received placebo or mBAS at initial processing (day 0) and at reimplanting (day 88) during the finishing period (day 0 to 178). Growth performance, health, cortisol concentration in tail-switch hair, and carcass variables were measured.

## 2 Materials and methods

This experiment was conducted at the Texas A&M – Beef Cattle Systems (College Station, TX, USA) from January to July 2024. Cattle were cared for in accordance with acceptable practices and experimental protocols approved by the Texas A&M AgriLife Research, Agriculture Animal Care and Use Committee (#2021-0308). Minimum, maximum, and average environmental temperatures during the experiment were -3.3, 36.1, and 21.6 °C, respectively, average humidity was 72.7 %, and a total of 779 mm of rain precipitation was observed.

The steers used in this experiment were the same that were used to evaluate mBAS (Ferappease®; FERA Diagnostics and Biologicals; College Station, TX, USA) during the receiving period whose results have been published (Pickett et al., 2024); thus, the receiving management are extensively explained in that manuscript. Briefly, 120 Angus-influenced steers were received and processed 125 days before the finishing period started. Upon receiving, steers were vaccinated (Vista Once SQ and Covexin 8; Merck Animal Health, Madison, NJ, USA), dewormed (Safe-Guard, Merck Animal Health), implanted (Synovex Choice®; Zoetis, Florham Park, NJ, USA), band-castrated (Callicrate Pro-BanderTM; No-Bull Enterprises, LLC St. Francis, KS, USA), and received a metaphylaxis treatment [2.5 mg/kg of body weight (**BW**) of Draxxin; Zoetis]. The receiving period lasted 60 days. After the receiving period, steers were offered a growing diet containing 69:31 concentrate to forage ratio (without receiving mBAS) for 65 days before the start of the finishing period, with the same arrangements of steers/pen as Pickett et al. (2024). At the end of this growing period, steers were loaded into 2 livestock trailers and transported for 1000 km (16 h) to simulate the stress of transport from a receiving/growing yard to a finishing yard (Samuelson et al., 2016). Steers returned to the same facility on day 0 and unloaded for the start of the finishing period.

### 2.1 Animals and treatments

**Fig. 1:**
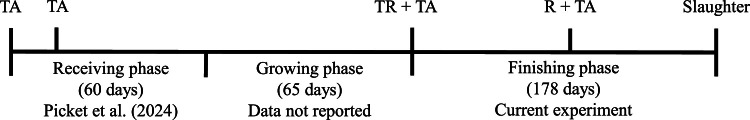
Comprehensive outline including the receiving period reported by Pickett et al. (2024), growing period (data not reported), and the finishing period described in this manuscript. Steers individually received 10 mL of the maternal bovine appeasing substance (Ferappease®; FERA Diagnostics and Biologicals; College Station, TX, USA) or mineral oil at initial processing (day 0) and at reimplanting (day 88; Synovex Plus®; Zoetis, Florham Park, NJ, USA) of the finishing period. Abbreviations mean; TA = treatment administration, TR = road transport (16 h), R = growth-promoting reimplant.

At initiation of the finishing period, steers were assigned to treatments in such a way that the same steers that received mBAS during the receiving period also received mBAS during the finishing period (Fig. 1). Treatments were as follows: 1) steers that topically received 10 mL of mineral oil on day 0 and 88 (**CON;** *n* = 50 steers), and 2) steers that topically received 10 mL of mBAS (Ferappease®; FERA Diagnostics and Biologicals) on day 0 and 88 (*n* = 57 steers). The initial BW of steers at the start of the experiment (day 0) were 340.0 ± 4 and 332.7 ± 5 kg for steers that received CON and mBAS, respectively. Steers were unloaded (day 0), sorted by treatment and immediately processed again for treatment administration, and then distributed to pens with the same arrangements of steers/pen and pen/treatment (5 pens/treatment) as in Pickett et al. (2024). On day 88 of the finishing period, steers received a terminal growth-promoting implant (Synovex Plus®; Zoetis). During treatment application on day 0 and 88, the CON pens were processed first followed by the mBAS pens to avoid cross-contamination.

Treatments (10 mL) were applied topically to the nuchal skin area (5 mL) and above the muzzle (5 mL). The active ingredient of mBAS is based on a proprietary mixture of fatty acids including palmitic, oleic, and linoleic acids, added at 10 % of the excipient and estimated to remain in treated animals for 15 days (Schubach et al., 2020). At the end of the finishing period (day 178), all steers were loaded into 2 commercial livestock trailers (3 pens/treatment in trailer 1 and 2 pens/treatment in trailer 2; pens loaded into different sections of the trailer) and transported to a packing facility (Tyson Foods; Amarillo, TX, USA) for slaughter on day 179. Throughout the finishing period, steers had free-choice access to water and total-mixed rations (**TMR**) that were offered once daily (0800 h) in a manner to yield 10 % residual orts (as-fed basis) and ensure ad libitum TMR intake. The feeding program during the finishing period consisted in an adaptation TMR (from day 0 to 9), transition TMR (from day 10 to 19) and finishing TMR (from day 20 to 178). Ingredient composition and nutrient characteristics are shown in Table 1. Treatment groups switched rows on day 0 and then every ∼30 days until slaughter to account for potential row effects.

**Table 1:** Composition and nutritional profile of the total mixed ration offered for ad libitum consumption to steers during the experiment.

Item	Adaptation (day 0 to 9)	Transition (day 10 to 19)	Finishing (day 20 to 178)
Composition, dry matter basis			
Cracked corn, %	42.6	52.5	61.7
Dried distillers’ grains, %	23.0	23.0	21.0
Bermudagrass hay, %	22.5	14.3	8.23
Liquid molasses, %	8.50	6.80	6.81
Mineral mix^a^, %	3.40	3.40	2.26
Nutritional profile,^b^ dry matter basis			
Net energy for maintenance, Mcal/kg	1.87	1.97	2.06
Net energy for gain, Mcal/kg	1.20	1.28	1.36
Neutral detergent fiber, %	24.9	23.6	15.7
Crude protein, %	14.2	13.7	13.6

a Containing 21 % Ca, 0.01 % P, 21 % NaCl, 0.20 % K, 0.10 % Mg, 0.045 % Cu, 0.001 % Se, 0.280 % Zn, 220,000 IU/kg of vitamin A, 19,800 IU/kg of vitamin D3, and 3500 IU/kg of vitamin E (Anipro Xtraperformance Feeds, College Station, TX, USA). Also contained sodium monensin (Rumensin; Elanco Animal Health, Greenfield, IN, USA) at 1320 g/metric ton.

b Wet chemistry procedures by a commercial laboratory (Dairy One Forage Laboratory, Ithaca, NY). Calculations for net energy for maintenance and gain used equations from the NASEM (2016). Steers were offered the growing diet from day -64 to -1, the step 1 diet from day 0 to 9, the step 2 diet from day 10 to 19, and the finishing diet from day 20 until day 178 (slaughter on day 179).

### 2.2 Sampling and laboratory analyses

The finishing TMR was collected every 2 weeks, whereas samples of the adaptation TMR and the transition TMR were collected every 5 days. Samples were pooled within TMR type and analyzed for nutrient content (Dairy One Forage Laboratory, Ithaca, NY, USA). All samples were analyzed by wet chemistry procedures for concentrations of crude protein (method 984.13; AOAC, 2006), and neutral detergent fiber using a-amylase and sodium sulfite (Van Soest et al., 1991; modified for use in an Ankom 200 fiber analyzer, Ankom Technology Corp.). Net energy for maintenance and gain were calculated using the equations proposed by NASEM (2016). The nutritional profile of TMRs is described in Table 1.

Steer shrunk BW was recorded immediately after arrival on day 0 (initial BW). Steer full BW was recorded on days 177 and 178, which were averaged and discounted a 4 % shrink (final BW). Steer average daily gain (**ADG**) was calculated using initial and final BWs. Feed intake [dry matter (**DM**) basis] was evaluated by recording daily TMR offer and measuring refusals every 7 days (day 0 to 178), divided by the number of steers within each pen, and expressed as kg per steer/day. Samples of the offered and non-consumed TMR were dried for 96 h at 50 °C in forced-air ovens to calculate DM. Gain to feed (**G:F**) ratio was calculated using total BW gain and total feed intake of each pen from day 0 to 178.

Steers were observed daily for health conditions (day 0 to 178) including signs of bovine respiratory disease as in Pickett et al. (2024). No signs of morbidity were observed during the finishing period, whereas 3 steers died due to bloat. Hair samples from the tail switch from each steer were collected on day 0 before treatment administration on that day, and then on day 15 (Schubach et al., 2017). Cortisol was extracted from hair samples, reconstituted in 100 μL of the buffer supplied with an ELISA cortisol kit (1-E3002; Salimetrics, LLC., State College, PA, USA), and stored at -80 °C as in Schubach et al. (2017). Samples were analyzed for cortisol concentrations using the ELISA kit (1-E3002; Salimetrics), with intra and inter-assay CV ≤ 6.5 %.

Hot carcass weight (**HCW**) was recorded upon slaughter on day 179, whereas trained personnel assessed carcass characteristics including backfat thickness at the 12th-rib, marbling, and *Longissimus muscle* (**LM**) area after a 24-h chill. Total final liveweight per pen was calculated by summing the final BW of steers within each pen, and the same approach was used for HCW of each pen (Mackey et al., 2024b; Pickett et al., 2024). Total liveweight gain per pen from the beginning of the receiving period until slaughter (day 179) used initial liveweight per pen from Pickett et al. (2024). A pen-basis economical evaluation was performed according to initial liveweight ($6.27 as in Pickett et al., 2024) and final liveweight of each pen ($4.27/kg), or according to total HCW of each pen ($6.87/kg of HCW). Values for final liveweight and HCW were obtained from the USDA Agricultural Marketing Services (5 area weighted cattle prices; https://www.ams.usda.gov) as 7/15/2024. Feed usage and costs by each pen was calculated using values from Pickett et al. (2024) for the receiving period ($385/metric ton), total feed intake of each pen during the 65 days when the growing diet was offered (5754 kg of DM for CON and 6336 kg of DM for mBAS; $370/metric ton), and total feed intake of each pen in this experiment ($365/ton for the adaptation TMR, $358/ton for the transition TMR, and $351/ton for the finishing TMR). Feed values were based on price of concentrate ingredients from a local supplier (Producers Cooperative Association; Bryan, TX, USA) during the experimental period (average January to July 2024), and cost of forage production by the research center (Texas A&M – Beef Cattle Systems). Medication cost used values from Pickett et al. (2024) as no medication was used during both growing and finishing periods. Profit of each pen was estimated as sale value – (initial value + feed costs + medication costs) on liveweight or HCW basis.

### 2.3 Statistical analysis

All data were analyzed using pen as the experimental unit, and Satterthwaite approximation to determine the denominator degrees of freedom for tests of fixed effects. Quantitative data were analyzed using the MIXED procedure of SAS (SAS Inst. Inc., Cary, NC, USA), whereas binary data were analyzed using the GLIMMIX procedure of SAS (SAS Inst. Inc.) with a binomial distribution and logit link function. The models for individual responses included pen(treatment) and steer(pen) as random variables, whereas models for pen-based responses used pen(treatment) as random variable (Bello et al., 2016). Model statements for BW parameters, G:F, and morbidity or mortality results contained the effects of treatment. Model statements for feed intake and hair cortisol contained the effects of treatment, day, and the resultant interaction. The specified term for all repeated statements was day, with pen(treatment) as subject for feed intake, and steer(pen) as subject for hair cortisol. The covariance structure used was first-order autoregressive, which provided the smallest Akaike information criterion and hence the best fit for all variables analyzed. All results are reported as least square means. Significance was set at *P* ≤ 0.05 and tendencies were determined if *P* > 0.05 and ≤ 0.10. Repeated measures are reported according to main treatment effect if the treatment × day interaction was *P* > 0.10.

## 3 Results and discussion

No treatment effect was detected (*P* = 0.86) for hair cortisol concentrations (Table 2), which is considered a biomarker of chronic stress (Burnett et al., 2014; Moya et al., 2015). Cortisol is gradually accumulated in the emerging tail hair, and its concentration represents long-term adrenocortical activity (Moya et al., 2013). Accordingly, Pickett et al. (2024) reported that mBAS administration reduced hair cortisol concentrations 14 and 28 days relative to initial processing. Schubach et al. (2020) administered mBAS at weaning and observed that hair cortisol concentrations were less in mBAS calves during the initial 14 days after weaning. In both studies, however, hair cortisol concentrations increased in cattle receiving mBAS or placebo upon the stress associated with weaning (Schubach et al., 2020) and feedlot arrival (Pickett et al., 2024). Hair cortisol concentrations were less (*P* < 0.01) across treatments on day 15 compared with day 0 of this experiment (3.13 and 3.85 pg/mg of hair, respectively; SEM = 0.15). Perhaps the stress elicited by the 16 h road transport was insufficient to affect hair cortisol concentrations, thus limiting the benefits of mBAS in alleviating this physiological response. Additional biomarkers of stress were not evaluated herein to test our hypothesis, such as plasma concentrations of acute-phase proteins and cortisol, because these require frequent handling of cattle for collection for proper assessment (Schubach et al., 2020). This experiment was designed to be representative of commercial finishing systems; hence, a minimal sampling schedule was designed to avoid disturbing steers and applying unnecessary handling stressors.

**Table 2:** Performance and hair cortisol responses during the finishing period (day 0 to 178) of beef steers administered the maternal bovine appeasing substance (**mBAS**; *n* = 5 pens) or mineral oil as placebo (**CON**; *n* = 5 pens)^a^.

Item	CON	mBAS	SEM	*P-value*
Initial BW,^b^ kg,	340.0	332.7	4.3	0.23
Final finishing BW,^b^ kg	565.7	572.0	6.6	0.51
Average daily gain, kg/day	1.271	1.342	0.025	0.04
Feed intake (dry matter basis),^c^ kg/day	9.45	9.25	0.24	0.57
Gain to feed,^d^ kg/kg	0.132	0.142	0.002	<0.01
Mortality,^e^ %	2.00	3.51	2.27	0.64
Removals,^e^ %	0.00	0.00	–	–
Hair cortisol,^f^ pg/mg of hair	3.47	3.51	0.15	0.86

a Steers individually received 10 mL of the mBAS (Ferappease®; FERA Diagnostics and Biologicals; College Station, TX, USA) or mineral oil (CON) at initial processing (day 0) and at reimplanting (day 88; Synovex Plus®; Zoetis, Florham Park, NJ, USA).

b Initial body weight (**BW**) was calculated based on shrunk BW recorded after a 16-h road-transport (day 0). Final BW was calculated by averaging steer full BW recorded on days 177 and 178 (immediately prior shipping to slaughter) and adjusted by a 4 % shrink.

c Feed intake was evaluated by recording daily offer and measuring refusals every 7 days from each pen, divided by the number of steers within each pen and expressed as kg per steer/day.

d Gain to feed was calculated using total BW gain (based on initial and final finishing BW), and total feed intake (kg of dry matter) of each pen during the 178-day finishing period.

e No steers were removed from the experiment, whereas mortalities were due to bloat.

f Hair samples were collected on day 0 before treatment administration on that day, and on day 15 as in Schubach et al. (2017). No treatment × day interaction was detected (*P* = 0.88).

Steer ADG was greater (*P* = 0.04) in mBAS compared with CON steers (Table 2), although such effect was not sufficient to alter (*P* ≥ 0.80) final BW or HCW (Table 3). Feed intake also did not differ (*P* = 0.57) between treatments, resulting in greater (*P* < 0.01) G:F of mBAS compared with CON steers (Table 2). No treatment differences were noted (*P* = 0.64) for mortality rate (Table 2), which was caused by bloat and likely unrelated to the treatments evaluated herein. Results from this experiment support our hypothesis, given that steer ADG was improved by mBAS but without an increase in feed intake after reimplanting (treatment × day interaction; *P* = 0.27). A day effect was observed (*P* < 0.01) for feed intake, but mostly caused by the expected decrease in intake during the final weeks of the finishing period (Silvestre et al., 2023). For both treatments, feed intake during the week before reimplant (week 11; 10.27 kg/day) did not differ (*P* = 0.36) compared to the week after reimplanting (week 12; 10.54 kg/day), and was less (*P* < 0.01) compared to week 13 (11.32 kg/day; SEM = 0.26). Therefore, the expected decrease in feed intake after reimplanting (Smith et al., 2020; Sperber et al., 2024) were not observed herein, preventing the potential benefits of mBAS in mitigating this outcome. The small-pen design of this experiment may explain the discrepancies in feed intake results after implanting compared with the large-pen studies by Smith et al. (2020) and Sperber et al. (2024). Processing events for reimplanting often takes longer and cattle are exposed to additional stressors in large-pen experimental designs (Grandin, 1980, 1998).

**Table 3:** Carcass parameters of beef steers administered the maternal bovine appeasing substance (**mBAS**; *n* = 5 pens) or mineral oil as placebo (**CON**; *n* = 5 pens) during a 178-day finishing period^a^^,^^b^.

Item	CON	mBAS	SEM	*P-value*
Hot carcass weight, kg	361.3	363.0	4.7	0.80
Dressing, %	63.8	63.4	2.5	0.24
Backfat, cm	1.50	1.48	0.10	0.87
*Longissimus muscle* area, cm^2^	86.6	85.8	1.06	0.59
Marbling score	428	429	12	0.94
Yield grade	3.20	3.22	0.11	0.90
Carcasses graded Choice or Prime, %	63.3	71.0	6.5	0.41

a Steers individually received 10-mL of the mBAS (Ferappease®; FERA Diagnostics and Biologicals; College Station, TX, USA) or mineral oil (CON) at initial processing (day 0) and at reimplanting (day 88; Synovex Plus®; Zoetis, Florham Park, NJ, USA). Steers were slaughtered on day 179 at a commercial packing facility (Tyson Foods; Amarillo, TX, USA).

b Trained personnel assessed carcass characteristics after a 24-h chill. Backfat thickness was measured at the 12th rib; marbling score: 300 = Slight^00^, 400 = Small^00^; yield grade calculated according to USDA (1997). Dressing percentage calculated as by dividing hot carcass weight and final body weight (average day 177 and 178) adjusted by a 4 % shrink.

The increase in ADG during the finishing period of mBAS steers was driven by their enhanced feed efficiency compared with CON steers. These outcomes are highly relevant to the feedlot industry, as production efficiency is key for profitability and sustainability of these systems (Wagner et al., 2014). Others have also reported increased G:F in cattle administered mBAS, which was associated with reduced stress-induced physiological and inflammatory reactions (Cappellozza & Cooke, 2022; Colombo et al., 2020; Fonseca et al., 2021). In turn, Cooke et al. (2025) administered mBAS to finishing steers at initial processing upon feedlot arrival, and 75 days later when steers were reimplanted. Steer ADG and feed intake were increased by mBAS administration, whereas G:F did not differ compared with steers receiving placebo (Cooke et al., 2025). Besides hair cortisol concentrations at the beginning of the finishing period, this experiment did not assess any other physiological responses to minimize cattle handling and represent industry management practices. Cooke et al. (2025) also did not collect blood or hair samples for physiological assessments. Hence, the biological mechanisms by which mBAS administration enhances ADG of finishing cattle, either by improving feed intake or G:F, requires further investigation.

No treatment effects were detected (*P* ≥ 0.24) for carcass quality traits, including dressing percentage, LM area, and marbling score (Table 3). Hence, the benefits of mBAS to steer finishing ADG and G:F were not translated into greater carcass quality (Retallick et al., 2013). Perhaps the increase in steer ADG was insufficient to improve carcass quality herein, such as marbling and HCW as reported by Cooke et al. (2025). Moreover, feed intake is positively associated with carcass quality in finishing cattle (Retallick et al., 2013), whereas mBAS administration improved feed intake in Cooke et al. (2025) but not in this experiment. Carcass dressing can be increased by mBAS administration to cattle during the week before slaughter (Mackey et al., 2024a); however, this approach was not included in our hypothesis and experimental design.

Pickett et al. (2024) compared pen-based liveweight change and final liveweight by the end of the 60-day receiving period, and reported a 61 % increase in total liveweight change and $1110 profit difference in pens housing mBAS cattle. The same approach was adopted herein to characterize pen-based productive and economical responses throughout the entire feeding period (feedlot receiving to slaughter), using the values from Pickett et al. (2024) for the receiving period (Table 4). The change in total liveweight/pen from was greater (*P* = 0.04) in mBAS vs. CON pens, resulting in greater (*P* ≤ 0.04) total liveweight/pen on day 178 and total HCW/pen upon slaughter. Total feed consumed per pen from tended to be greater (*P* = 0.08) in mBAS pens, whereas G:F was greater (*P* = 0.05) in mBAS vs. CON pens. These results were caused by decreased removal rate during the receiving period (Pickett et al., 2024) and increased ADG and G:F of mBAS steers in this experiment. The improved growth performance of mBAS steers herein can be attributed to residual health benefits from mBAS during receiving (Cooke et al., 2017; Galyean et al., 2022), and the direct effects of mBAS administration during the finishing period.

**Table 4:** Productive and economical responses during the entire feeding period (feedlot receiving to slaughter) of pens containing beef steers administered the maternal bovine appeasing substance (**mBAS**; *n* = 5 pens) or mineral oil as placebo (**CON**; *n* = 5 pens)^a^.

Item	CON	mBAS	SEM	*P-value*
Productive responses				
Initial liveweight,^b^ kg/pen	2175	2178	2	0.47
Final liveweight,^b^ kg/pen	5544	6292	210	0.03
Liveweight gain, kg/pen	3369	4114	203	0.04
Hot carcass weight, kg	3540	3992	126	0.04
Total feed intake (dry matter basis),^c^ kg/pen	25,791	27,941	761	0.08
Gain to feed,^d^ kg/kg per pen	0.130	0.147	0.005	0.05
Economical assessment^e^				
Initial value, $/pen	14,185	14,202	15	0.47
Sale value, $/pen				
Liveweight	23,841	27,055	874	0.03
Hot carcass weight	24,348	27,455	890	0.04
Feed cost, $/pen	9284	10,059	274	0.08
Medication cost, $/pen	265	229	30	0.53
Profit, $/pen				
Liveweight	-43.2	2388	725	0.04
Hot carcass weight	614	2958	744	0.05

a Steers individually received 10-mL of the mBAS (Ferappease®; FERA Diagnostics and Biologicals; College Station, TX, USA) or mineral oil (CON) at initial processing and 14 days later during the receiving period (Pickett et al., 2024). Treatments were reapplied at initial processing (day 0) and at reimplanting (day 88; Synovex Plus®; Zoetis, Florham Park, NJ, USA). Steers were slaughtered on day 179 at a commercial packing facility (Tyson Foods; Amarillo, TX, USA).

b Initial liveweight from Pickett et al. (2024); final liveweight = sum of the averaged steer full body weight (average day 177 and 178) recorded before slaughter of each pen discounted a 4 % shrink; hot carcass weight = sum of the hot carcass weight of steers from each pen.

c Evaluated by recording daily offer and measuring refusals from each pen feedlot receiving (Pickett et al., 2024) to slaughter. Total feed used per pen during the 65 days when the growing diet was offered was 5754 kg of DM for CON and 6336 kg of DM for mBAS. Reported in dry matter basis.

d Feed efficiency was calculated using liveweight gain and total feed intake (kg of dry matter) of each pen from feedlot receiving (Pickett et al., 2024) to slaughter.

e Initial value was calculated using pen shrunk liveweight adjusted by a 4 % shrink as $6.27/kg. Sale value based on pen final liveweight as $4.27/kg, or as $6.87/kg for hot carcass weight. Feed cost was estimated at (dry matter basis) $385/ton for the receiving diet (Pickett et al., 2024), $370/ton for the growing total-mixed ration (**TMR**), $365/ton for the adaptation TMR, $358/ton for the transition TMR, and $351/ton for the finishing TMR. Medication cost as described in Pickett et al. (2024). The profit of each pen was estimated as final value – (initial value + feed costs + medication costs).

The mBAS pens had greater (*P* ≤ 0.04) final value as liveweight or HCW basis, with a tendency (*P* = 0.08) for increased feed costs and similar (*P* = 0.53) medication costs compared with CON pens (Table 4). Accordingly, profit for mBAS pens was greater (*P* ≤ 0.05) compared with CON pens on liveweight and HCW basis, which resulted in return-on-investments (**ROIs**) of ≥1698 %. The ROIs were calculated according to profit differences of CON and mBAS pens based on liveweight ($-43.2 vs. $2388 = $2431) or HCW ($614 vs. $2958 = $2344), and divided by $138 for mBAS costs ($3 for each individual 10 mL dose; 24 doses/pen during the receiving period, 11.2 doses/pen on day 0, and 10.8 dose/pen on day 88 due to removals and mortalities). These pen-based assessments provide evidence of the economic benefits of mBAS administration throughout the feeding period, but should be interpreted with caution as values used for cattle, feed, and medications vary according to time and location.

## 4 Conclusions

The hypothesis that applying mBAS to feedlot steers during the stressful events within the finishing period (upon arrival and at the time of reimplanting) would reduce stress responses was not confirmed, since the stress variables measured herein were not modified. Nonetheless, improved growth performance (ADG and feed efficiency) was observed in steers that received mBAS. Our experimental design does not provide a clear explanation for this improvement in production efficiency, which is a valuable trait for the feedlot industry. Hence, research is still warranted to further investigate the effects of mBAS as an adjuvant to routine management procedures during the finishing period.

## Financial support statement

No funding to report.

## Data availability

None of the data or the models were deposited in an official repository.

## Ethical statement

All animals were cared for in accordance with acceptable practices and experimental protocols reviewed and approved by the Texas A&M AgriLife Research, Agriculture Animal Care and Use Committee (#2021-0308).

## CRediT authorship contribution statement

**Shea J. Mackey:** Writing – original draft, Visualization, Methodology, Investigation, Data curation, Conceptualization. **Reinaldo F. Cooke:** Writing – original draft, Visualization, Supervision, Project administration, Methodology, Investigation, Funding acquisition, Formal analysis, Conceptualization. **Izadora S. de Souza:** Writing – review & editing, Investigation. **Autumn T. Pickett:** Writing – review & editing, Investigation.

## Declaration of competing interest

All authors of this manuscript have no conflict of interest to report.
